# Effects of Dietary Valine Levels on Production Performance, Egg Quality, Antioxidant Capacity, Immunity, and Intestinal Amino Acid Absorption of Laying Hens during the Peak Lay Period

**DOI:** 10.3390/ani11071972

**Published:** 2021-06-30

**Authors:** Huafeng Jian, Sasa Miao, Yating Liu, Huaiyu Li, Wenting Zhou, Xiaoming Wang, Xinyang Dong, Xiaoting Zou

**Affiliations:** Institute of Feed Science, College of Animal Science, Zhejiang University, Hangzhou 310058, China; jianhuafeng@zju.edu.cn (H.J.); 21917066@zju.edu.cn (S.M.); 22017064@zju.edu.cn (Y.L.); 21817082@zju.edu.cn (H.L.); 21817022@zju.edu.cn (W.Z.); 22017083@zju.edu.cn (X.W.); sophiedxy@zju.edu.cn (X.D.)

**Keywords:** Fengda No.1 laying hens, valine, antioxidant enzymes, serum free amino acids, neutral amino acid transporters

## Abstract

**Simple Summary:**

In the past, many studies have been carried out to investigate the effect of dietary valine supplementation on laying hens’ production performance and egg quality. However, knowledge concerning the optimal valine requirement during the peak lay period is limited and mainly restricted to the production performance of the hens. Within this context, the present study aimed to assess the impact of dietary valine levels on production performance, egg quality, antioxidant capacity, and immunity, as well as on intestinal amino acid absorption (i.e., serum free amino acids, digestive enzymes, and amino acid transporters). Dietary valine supplementation exerts positive effects on the production performance of the laying hens by promoting amino acid nutrient uptake and utilization. However, dietary valine supplementation might inhibit the absorption of dietary protein by downregulating peptide transporter expression of the small intestine, eventually resulting in the reduction of egg quality.

**Abstract:**

The present study aimed to assess the impact of dietary valine levels on layer production performance, egg quality, immunity, and intestinal amino acid absorption of laying hens during the peak lay period. For this purpose, a total of 960 33-week-old Fengda No.1 laying hens were randomly divided into five experimental groups and fed with valine at the following different levels in a feeding trial that lasted 8 weeks: 0.59, 0.64, 0.69, 0.74, and 0.79%, respectively. Productive performances were recorded throughout the whole rearing cycle and the egg quality, serum indexes, and small intestine transporters expression were assessed at the end of the experiment after slaughter (41 weeks) on 12 hens per group. Statistical analysis was conducted by one-way ANOVA followed by LSD multiple comparison tests with SPSS 20.0 (SPSS, Chicago, IL, USA). The linear and quadratic effects were tested by SPSS 20.0. Egg mass, laying rate, broken egg rate, and feed conversion ratio were significantly improved with increasing dietary valine levels. However, the egg weight, eggshell thickness, albumen height, Haugh unit, and egg yolk color were significantly decreased with increasing dietary valine levels. Serum catalase (CAT), immunoglobulin A (IgA) and IgM levels, and malondialdehyde (MDA) levels were negative responses to valine-treated laying hens. Dietary supplemented valine enhanced the trypsin activity of duodenum chime and promoted the mRNA expression levels of ATB^0,+^, and LAT4 in the jejunum and corresponding serum free Ile, Lys, Phe, Val, and Tyr level. However, valine treatment significantly downregulated the mRNA expression levels of PePT1, B^0^AT1, LAT1, and SNAT2 in the small intestines and corresponding serum free Arg, His, Met, Thr, Ala, Asp, Glu, Gly, and Ser level. Our results suggest that 0.79% valine dietary supplementation can improve production performance by promoting amino acid nutrient uptake and utilization, and suggest a supplement of 0.79% valine to diet.

## 1. Introduction

Diet composition improvement represents a key factor to enhance the health status and welfare of animals, as well as to enhance productivity in livestock [1,2,3,4]. Essential amino acid (EAA) refers to amino acids that cannot be synthesized by the body and must be provided through diet. Valine (Val) is a branched-chain amino acid (BCAA, also including leucine and isoleucine) [5]. As an EAA, valine participates in the synthesis of protein and is a precursor of other amino acids, or it takes part in glucose metabolism as glucogenic amino acid [6,7]. Valine is the 5th limiting amino acid of laying hens after methionine (Met), lysine (Lys), tryptophan (Trp), and threonine (Thr) [8]. For poultry, a supplement of adequate valine is necessary for maintaining higher productive performance, and an excess or deficiency of valine could result in antagonism among BCAAs [9]. NRC (1994) suggested 0.70% valine in the basal diet is required for commercially laying hens during the peak lay period [10], while China’s “Chicken Feeding Standard (NY/T33-2004)” suggested that 0.59% valine is required [11]. The requirement of valine in different commercial laying hens has been well evaluated, including in Hy-Line W-36 laying hens [12,13], Lohmann Brown hens [14], and Dekalb Brown laying hens [8]. The total valine requirement of small-framed first-cycle laying hens (41 to 60 weeks of age) was 597.3 mg/d based on the egg mass of Hy-Line W-36 laying hens [15]. Arginine (Arg), histidine (His), isoleucine (Ile), leucine (Leu), lysine (Lys), Met, phenylalanine (Phe), Thr, Trp, and Val are necessary for egg production in the free amino acid diet of laying hens, and glutamic acid (Glu) is essential for maximum egg production [16].

The small intestines are the main absorption site of dietary amino acids. In the small intestine, the transportation of amino acids is mainly mediated by the amino acid transporter in the intestinal epithelial cells. These molecular transporters mainly express on the cellular membranes and play a vital role in the cellular uptake of amino acids [17]. PepT1 (peptide-transporter 1), a peptide transporter, can transport di- and tripeptides from the intestinal lumen into enterocytes [18]. B^0^AT1 (B^0^-type amino acid transporter 1) is an Na^+^-independent transporter of neutral amino acid and responsible for the uptake of neutral amino acids such as Met, Leu, Ile, and Val [19]. SNAT2 (sodium-coupled neutral amino acid transporter 2), an Na^+^-coupled neutral amino acid transporter, is mainly expressed in the small intestine [20]. ATB^0,+^ (amino acid transporter B^0,+^), is an Na^+^-dependent neutral and cationic amino acid transporter [21]. LAT1 and LAT4 (L-type amino acid transporters) are Na^+^-dependent neutral amino acid transporters and responsible for the majority of cellular Leu uptake [22]. In the intestine, LAT4 is mainly present in the cells of the crypt and expressed in the basolateral membrane [23]. In chicken, PepT1 mRNA was higher in the duodenum, while B^0^AT and LAT1 mRNA were higher in the ileum after 14 days post-hatching [24]. It is well known that the expression levels of LAT1, LAT4, and SNAT2 represent their capacity to absorb and transport BCAAs [25]. However, many detrimental factors, including oxidative injury and amino acid antagonism, can affect the expression of amino acid transporters, and result in negative effects on productive performance. For example, lipid peroxidation and reactive oxygen species (ROS) generation can significantly inhibit the transportation of amino acids by enterocyte brush border membrane vesicles [26,27].

In China, due to increased health awareness and spending power, consumers are increasingly demanding egg quality, and it is popular to purchase eggs produced by local laying hens. With the constant increase in demand for egg quality by consumers in China, and to meet the needs of consumers, a large number of the local breeds of laying hens are farmed. However, little scientific information is available about the optimal requirement of dietary valine for laying hens of the local breeds in China during the peak lay period. Fengda No.1 laying hens are a Chinese local breed, mainly present in eastern China. The number of Fengda is more than 10 million birds in China [28,29]. However, there has been no report about the optimal valine requirement of Fengda No.1 laying hens during the peak lay period. Thus, we hypothesized that the optimal level of valine could maximize the production performance of Fengda No.1 laying hens during the peak lay period by improving antioxidant capacity, immunity, and intestinal absorption function. Therefore, the aim of the present study is to evaluate the effects of dietary valine levels on production performance, egg quality, antioxidant capacity, immunity, and intestinal digestion and absorption of Fengda No.1 laying hens during the peak lay period.

## 2. Materials and Methods

### 2.1. Diets, Birds, and Management

Corn and soybean meal were selected as major ingredients to make up a corn-soybean-type basal diet and prepared according to NRC (1994) [10] and China’s Chicken Feeding Standard (2004) [11]. Synthetic L-Val (98% purity, Specom Biochemical Co. Ltd., Zhangjiagang, China) was supplemented to the basal diet in 0%, 0.0508%, 0.1016%, 0.1523%, and 0.2031% increments, resulting in experimental diets containing 0.59, 0.64, 0.69, 0.74, and 0.79% of valine, respectively (Table 1). In addition, the ratio of other amino acids in the diet was corrected to be consistent with each group according to dietary protein.

A total of 960 33-week old healthy Fengda No.1 laying hens with similar BW and laying rate were randomly allocated into 5 experimental groups, and each group included 6 replicates of 32 laying hens (8 birds/cage). The average BW of each group was 1528, 1505, 1563, 1579, and 1603 g, respectively. This study lasted 9 weeks, including a one-week acclimation period and 8-week experimental period. All hens were housed in an environmentally controlled room in which the temperature was maintained at approximately 23 °C. The hens were exposed to a 16 h photoperiod throughout the experiment by the use of artificial lighting. Hens were supplied with water and fed a complete feeding mixture. Over the 8-week experimental period, laying hens were visually inspected at least twice daily. At the morning check, eggs were collected, and feed provided at approximately 7 a.m. A follow-up check and feed were provided at 2 p.m. each day. All hens received and consumed about 100 g of diet per bird each day, and they had ad libitum access to fresh water. All animal works in this experiment were conducted following the Chinese Guidelines for Animal Welfare and approved by the Zhejiang University Institutional Animal Care and Use Committee (No. ZJU2013105002) (Hangzhou, China).

### 2.2. Productive Performance and Determination of Egg Quality

Egg numbers, egg weight, broken egg, soft shell egg, and mortality were recorded daily, whereas the feed intake and feed conversion ratio (FCR) were calculated to correspond with 7-day feed manufacture periods. At the end of the 8-week experiment, 30 eggs from each group (5 eggs per replication, 6 replications per group) were randomly collected from each group. A total of 150 eggs were used to assess egg quality. Eggs were weighed and cracked. Albumen height, Haugh units, yolk color, and eggshell strength were measured with a digital egg tester (DET-6000, NABEL, Kyoto, Japan). Eggshell thickness (without the shell membrane) was measured at the sharp end, equator, and blunt end parts of the egg, using an eggshell thickness gauge (Robotmation Co., Ltd., Kyoto, Japan).

### 2.3. Sample Collection and Processing

The parameters of serum antioxidant, free amino acids, immunity, duodenal digestive enzyme, and intestinal transporter mRNA expression levels were studied after the experiment finished. At the end of the 8-week experiment, 2 hens were randomly selected from each repeat (12 hens in each group; a total of 60 hens) and fasted for 12 h. A blood sample (5 mL, bird-1) was collected from the vein under the wing using a pro-coagulant tube (Jiangsu Kangjie Medical Devices Co., Ltd., Jiangyan, China) at 10 a.m. After centrifugation of the blood at 3000× *g* for 10 min, serum was separated and stored in 1.5 mL Eppendorf tubes at −80 °C. Before analysis, the serum was thawed at 4 °C. After blood sampling, hens were euthanized with pentobarbital sodium and sacrificed. The duodenum contents, the intestine segments of the duodenum, jejunum, and ileum were carefully collected, immediately placed in cryogenic vials, and stored at −80 °C until they were processed for digestive enzymes and mRNA expression.

### 2.4. Serum Index and Digestive Enzyme Assays

Three days later, the activities of total antioxidative capacity (T-AOC), total superoxide dismutase (T-SOD), glutathione peroxidase (GSH-Px), catalase (CAT), and concentrations of malondialdehyde (MDA) in the serum were measured using commercial kits (Nanjing Jiancheng Bioengineering Institute, Nanjing, China). Trypsin, lipase, and α-amylase in the duodenum contents were measured using commercial kits (Nanjing Jiancheng Bioengineering Institute, Nanjing, China). The optical density (OD) value of each sample was measured by spectrophotometer (UV-1601 UV–VIS Spectrophotometer, Shimadzu Corporation, Tokyo, Japan). Serum immunoglobulins (Ig), including IgA, IgG, and IgM, were measured using the same batch number of chicken-specific ELISA quantitation kits (Nanjing Jiancheng Bioengineering Institute, Nanjing, China), respectively. The tolerance within batch and the tolerance between batches of IgA, IgG, and IgM ELISA kits were <10% and 12%. According to the instructions of the manufacturer, the per sample was analyzed 3 duplicates, and the absorbance was measured at 450 nm. The concentrations of IgA, IgG, and IgM were calculated by using standard curves constructed from the standards run on the plate. A microplate reader (Biotek ELX800; Biotek Instruments, Inc., Winooski, VT, USA) was used in the determination. All assays were performed according to the manufacturer’s instructions.

### 2.5. Determination of Serum Free Amino Acids

Serum was deproteinized by mixing one volume of serum and four volumes of sulfonic acid (5%), vortexed (30 s), and centrifuged for 30 min at 18,000× *g*. Subsequently, a 20 μL aliquot of the supernatant was in a high-performance liquid chromatography column (Hitachi L-8900 Amino Acid Analyzer, Hitachi High Technologies Japan, Inc. Tokyo, Japan). Amino acids were separated by cation exchange using lithium buffers, with the UV light detection (570 nm) of individual amino acids (440 nm for proline) performed by post-column ninhydrin derivatization.

### 2.6. Total RNA Extraction and Real-Time PCR

The intestinal mRNA expression levels of PepT1 (SLC15A1), B^0^AT1 (SLC6A19), SNAT2 (SLC38A2), ATB^0,+^ (SLC6A14), LAT1 (SLC7A5), and LAT4 (SLC43A2) were determined using real time-PCR. Total RNA was extracted using TRIzol reagent (Takara code: 9109, Shiga, Japan). RNA quality and quantity were determined using a NanoDrop 2000 spectrophotometer (Thermo Fisher Scientific, Waltham, MA, USA). cDNA was synthesized with a HiScriptIIqRT SuperMix Reverse Transcriptase (Vazyme Biotechnology, Nanjing, Jiangsu, China) according to the manufacturer’s instructions. Briefly, 1 μg total RNA was used to erase gDNA at 42 °C for 2 min. The reverse transcription was conducted at 50 °C for 15 min and 85 °C for 5 s. Real-time PCR was performed on a CFX96TM Real-Time System (Bio-Rad, Hercules, CA, USA) in triplicate, in a total volume of 20 μL consisting of 10 μL SYBR Premix PCR kit (Vazyme Biotechnology, Nanjing, Jiangsu, China), 0.5 μL each of primer (10 μM), 2 μL of cDNA template, and 9 μL double-distilled water. The PCR cycle conditions were 95 °C for the 30 s, followed by 40 cycles of 95 °C for 5 s and 60 °C for 30 s. Melting curve analysis was used to confirm the specificity and reliability of PCR products. There were 6–8 samples in each group, each sample was conducted in duplicate, and no template control was included. β-actin was used as a house-keeping gene to normalize target gene levels. The relative mRNA expression was calculated using the 2^−^^△△Ct^ method. Premiers used in this study were designed with Premier 5.0 (Table 2) and synthesized in Tsingke (Hangzhou, China).

### 2.7. Statistical Analysis

The data was collected by MS Excel 2019. The Gaussian distribution of data was analyzed by the Normality test (SPSS 20.0). The variance of the data was analyzed by the homogeneity of variance test (SPSS 20.0). Statistical analysis was performed with one-way ANOVA followed by LSD multiple comparison tests with SPSS 20.0 (SPSS, Chicago, IL, USA). Linear and quadratic effects were tested by SPSS 20.0 and considered significant at *p* < 0.05, or considered a trend at 0.05 ≤ *p* < 0.10. Data are presented as means and SEM and are considered significant at *p* < 0.05, or considered a trend at 0.05 ≤ *p* < 0.10. Quadratic regression (Y = c + bx + ax2) was fitted by SPSS 20.0 to determine the linear and quadratic effects of valine concentration on the laying rate. GraphPad Prism 9 (GraphPad Software Inc., San Diego, CA, USA) was used for graphical presentations.

## 3. Results

### 3.1. Laying Hens Performance

As shown in Table 3, egg mass and laying rate showed linear or quadratic increases with the increasing of dietary valine levels (*p* < 0.05). However, egg weight, broken egg rate, and feed conversion ratio (FCR) were significantly decreased (*p* < 0.01), whereas it had no effect on feed intake. The optimal dietary valine concentration that maximized the laying rate in Fengda No.1 laying hens was 0.79% according to quadratic regression analysis (Y = 81.451 + 0.293X + 0.019X^2^; R^2^ = 0.004, *p* = 0.026).

### 3.2. Egg Quality

As shown in Table 4, we found that eggshell thickness (sharp end), albumen height, Haugh unit, and egg yolk color showed a linear or quadratic decrease after valine treatments (*p* < 0.05). The egg weight presented a decreasing trend with the increasing of dietary valine levels (0.05 < *p* < 0.1). There was no significant difference in eggshell strength and the blunt end of eggshell thickness among all experimental groups (*p* > 0.05).

### 3.3. Serum Antioxidant Capacity

The effects of dietary valine levels on the antioxidative enzyme activities of serum are summarized in Table 5. The activities of serum CAT showed a significant decrease in a quadratic manner with increasing of dietary valine levels (*p* < 0.05), whereas the activities of T-AOC and MDA levels were significantly increased (*p* < 0.05). There is no significant difference among all groups in serum T-SOD and GSH-Px activity (*p* > 0.05).

### 3.4. Serum Immunoglobulins Levels

**Figure 1:**
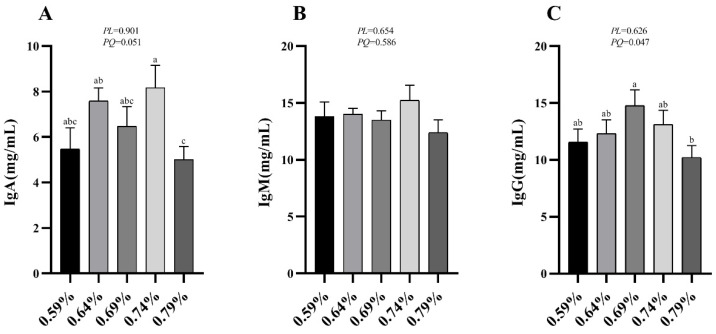
Effects of dietary valine levels on serum immune indices of laying hens. Values are represented as the mean and SEM (n = 8). Means with different superscript letters (a–c) within a column differ significantly (*p* < 0.05). IgA, Immunoglobulin A; IgM, Immunoglobulin M; IgG, Immunoglobulin G.

The effects of dietary valine levels on immune indices in serum are summarized in Figure 1. The results indicated that the serum IgA and IgM show a significantly quadratic decrease as dietary valine levels increase (*p* < 0.05) (Figure 1A,C). No significant difference was observed among all groups in serum IgM level (Figure 1B).

### 3.5. Digestive Enzymes Activities

**Figure 2:**
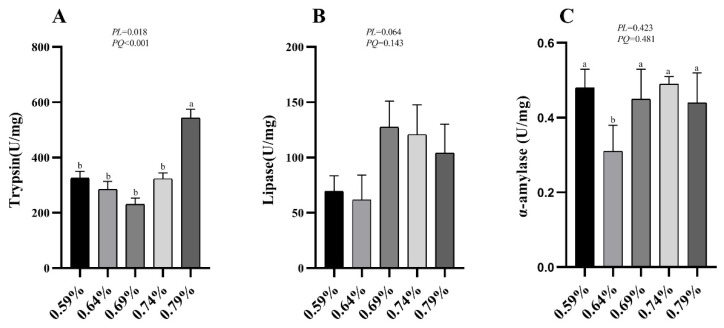
Effects of dietary valine levels on activities of duodenum digestive enzymes of laying hens. Values are represented as the mean and SEM (n = 8). Means with different superscript letters (a, b) within a column differ significantly (*p* < 0.05).

As shown in Figure 2A, the activity of trypsin showed a significant increase with the increasing of dietary valine levels (*p* < 0.05), and the lipase activity showed an increasing trend (0.05 < *p* < 0.10). No significant difference was observed in the activity of α-amylase among all treatments (*p* > 0.05).

### 3.6. Serum Free Amino Acids

The effects of dietary valine levels on the serum free amino acids of laying hens are presented in Table 6. As shown in Table 6, serum free Ile, Lys, Phe, Val, and Tyr showed a quadratic increase with the increasing of dietary valine levels (*p* < 0.05), whereas serum free Arg, His, Met, Thr, Ala (Alanine), Asp (Aspartic acid), Glu, Gly (Glycine), and Ser (Serine) were significantly decreased (*p* < 0.01). In addition, the concentration of serum Leu showed a linear decrease as the dietary valine levels increased (*p* < 0.05). No difference was observed in serum free Pro (proline) and Cys (cystine) among all treatment groups (*p* > 0.05).

### 3.7. Gene mRNA Expression Levels of PepT1, B^0^AT1, SNAT2, ATB^0,+^, LAT1 and LAT4

**Figure 3:**
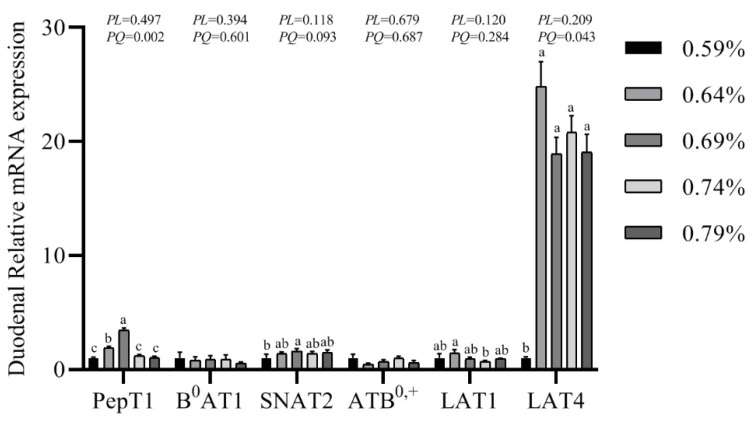
Effects of dietary valine levels on mRNA expression levels of PepT1, B^0^AT1, SNAT2, ATB^0,+^, LAT1, and LAT4 in the duodenum of laying hens. Values are represented as the means and SEM (n = 6–8). Means with different superscript letters (a–c) within a column differ significantly (*p* < 0.05).

**Figure 4:**
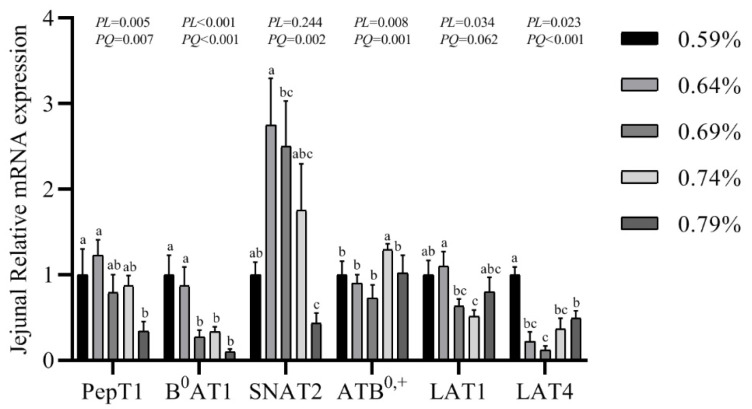
Effects of dietary valine levels on mRNA expression levels of PepT1, B^0^AT1, SNAT2, ATB^0,+^, LAT1, and LAT4 in jejunum of laying hens. Values are represented as the mean and SEM (n = 6–8). Means with different superscript letters (a–c) within a column differ significantly (*p* < 0.05).

**Figure 5:**
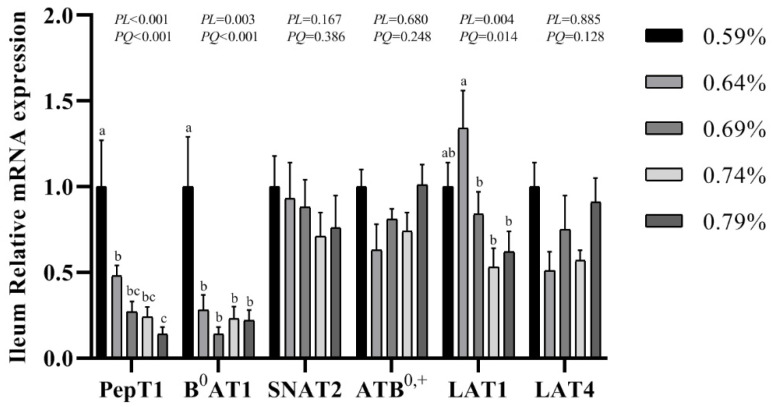
Effects of dietary valine levels on mRNA expression levels of PepT1, B^0^AT1, SNAT2, ATB^0,+^, LAT1, and LAT4 in ileum of laying hens. Values are represented as the mean and SEM (n = 6–8). Means with different superscript letters (a–c) within a column differ significantly (*p* < 0.05).

The gene expression of transporters in the small intestine are shown in Figure 3, Figure 4 and Figure 5. In the duodenum, the mRNA levels of PePT1 and LAT4 showed a significantly quadratic decrease as the dietary valine levels increased (*p* < 0.05), whereas this had no effects on the mRNA levels of B^0^AT1, SNAT2, ATB^0,+^, and LAT1 (Figure 3, Figure 4 and Figure 5). In the jejunum, valine treatment significantly downregulated the mRNA levels of PePT1, B^0^AT1, and SNAT2, whereas it significantly upregulated the mRNA levels of ATB^0,+^, and LAT4 in a linear or quadratic manner. The expression level of LAT1 presented a decreasing trend in the jejunum (Figure 3, Figure 4 and Figure 5). In the ileum, valine treatment significantly downregulated the mRNA levels of PePT1, B^0^AT1, and LAT1 in a linear or quadratic manner, but did not affect the mRNA expression levels of SNAT2, ATB^0,+^, and LAT4 (Figure 3, Figure 4 and Figure 5).

## 4. Discussion

The current study evaluated the effects of dietary valine levels on production performance, egg quality, antioxidant capacity, immune function, and intestinal amino acid absorption in the peak lay period of hens. After being fed eight weeks of different levels of valine diet, we found egg mass and laying rate showed a significant increase with the. increasing of dietary valine levels, and valine treatment significantly reduced FCR and broken egg rate. This is consistent with the previous reports that egg production gradually increased as supplemented dietary valine concentration increased from 0.525% to 0.765% or from 0.515% to 0.865% in 39-to-46-week- or 41-to-60-week-old Hy-Line W-36 laying hens [12,15]. It has been demonstrated that valine is necessary for egg protein synthesis, and valine deficiency could result in the reduction of egg production [13]. The optimal dietary valine level that maximized the laying rate in Fengda No.1 laying hens was 0.79% according to quadratic regression analysis (Y = 81.451 + 0.293X + 0.019X^2^; R^2^ = 0.004, *p* = 0.026). However, valine treatment significantly decreased the average egg weight with the increasing of dietary valine levels, which is inconsistent with the previous reports [8,12,14,15]. Previous reports indicated that egg weight was decreased as dietary valine concentration decreased from 0.865% to 0.515% or from 0.765% to 0.525% [8,15]. Eder and Peganova’s experiment showed that egg weight increased as dietary digestible valine concentration increased to 0.74%, but when the dietary valine level increased to 0.80% and 0.86%, egg weight significantly decreased, which is consistent with Harms and Russell’s report [12,14]. Interestingly, we found that there no significant difference was observed in the feed intake among all treatments in the current study, which is inconsistent with those reported previously [8,12,14,15]. The previous report indicated that average feed intake was increased from 66.5 g/hen/d to 92.9 g/hen/d as valine concentration increased from 0.515% to 0.725% [15]. Likewise, as the dietary valine concentration increased from 0.525% to 0.765%, the average feed intake was increased from 81.2 g/hen/d to 96.2 g/hen/d in Hy-Line W-36 laying hens [12]. Differences in macro ingredient contents (peanut meal vs. soybean meal) and crude protein levels of the basal diet may explain these feed intake differences.

We found that the sharp end of eggshell thickness, albumen height, Haugh unit, and egg yolk color showed a linear or quadratic decrease after valine treatment. This is in contrast to the results that dietary valine did not affect the relative yolk, relative shell weight, and relative albumen weight when digestible valine concentration varied from 0.555% to 0.666% in laying hen diets [8]. Studies have demonstrated the lowest eggshell thickness in a 0.585% valine diet, rather than in the highest 0.865% valine diet [15]. In addition, the previous studies found that Haugh unit was affected by diet crude protein levels, and low crude protein usually showed higher Haugh unit values [30]. These differences may be associated with the significantly increased laying rate. In this study, eggshell strength is not affected by valine treatment. Eggshell strength is an important indicator to evaluate egg quality, which is affected by several factors, such as dietary Ca and P levels [31].

Oxidative stress refers to metabolic and radical substances or so-called reactive (oxygen, nitrogen, or chlorine) species [32]. Recently, several studies have reported that dietary amino acid supplementation improved body antioxidant enzyme activity such as L-tryptophan [33] and L-threonine [34]. Our results showed that the serum CAT was quadratically decreased as the dietary valine levels increased, while the T-AOC activity was significantly increased. However, previous studies have demonstrated that excess supplemented valine did not affect the activities of T-AOC and MDA in serum or liver [35]. We found valine treatment did not affect the activities of serum T-SOD and GSH-Px. In addition, we found that the MDA level was significantly increased with the increasing of dietary valine levels. MDA has been demonstrated to endogenously reflect lipid peroxidation, which is the consequence of diminished antioxidant protection as concentrations of reactive oxygen species (ROS) increase [33]. Oxidative stress and lipid peroxidation, induced by high productive performance, may explain these antioxidant enzymes’ activity reduction and increased MDA levels. Valine not only participates in protein synthesis but also affect the production of immunoglobulins [35]. We found that the serum IgA and IgM levels were significantly decreased with the increasing of dietary valine concentration, while they did not affect serum IgM level. However, excess supplemented dietary isoleucine did not affect the serum concentrations of IgG, IgA, or IgM in the laying hens [36]. It has been reported that supplemented amino acids such as L-threonine or L-tryptophan significantly increased serum IgG and IgM concentrations in laying hens [33,34]. These results indicate that the immunity reduction of laying hens may be caused by higher production performance during the peak lay period.

The small intestine is the main organ for nutrient digestion and absorption for domestic animals [37]. Digestive enzymes, including amylase, protease, and lipase, are mainly secreted from the pancreas, and distributed and activated in the duodenal and jejunal sections of the small intestine for nutrient digestion and absorption in poultry [38]. With the increase of dietary valine levels, the activities of trypsin and lipase showed linear or quadratic increases. In broiler chickens, supplemented threonine did not affect the activities of amylase, pepsin, or trypsin, which is inconsistent with our current experiment results [39]. Trypsin and its activity play a very important role in amino acid digestibility. Diet supplementation of L-threonine had no effect on the activity of digestive enzymes in laying hens in a high-temperature-and-humidity environment [34]. In the current study, the activities of trypsin and lipase were increased in a higher concentration in valine diets, which may be helpful in the digestion of amino acids and could maintain a higher production performance of laying hens. Zhang et al. reported that dietary yeast culture supplementation significantly increased chymotrypsin and α-amylase activities of duodenal chyme, and together with upregulation of intestinal-health-related gene expression, improved production performance [40].

Arg, His, Ile, Leu, Lys, Met, Phe, Thr, Trp, and Val are the essential amino acids of egg formation [16]. Our results suggest that serum free Ile, Lys, Phe, Val, and Tyr were significantly increased. In laying hens, plasma Thr was increased with increasing dietary Thr levels [41]. Dietary supplemented excess L-Val significantly increased serum free Val as L-Val concentration increased [35]. Serum free Thr showed a quadratically significant increase as supplemental Thr increased in laying hens, which is consistent with our results [34]. In peak laying hens, a diet supplemented with a low dose of melatonin (0.625 and 2.5 mg MEL/kg diets) significantly improved serum free amino acid levels such as Asp, Thr, Ser, Glu, Gly, Ala, Ile, Leu, Tyr, Phe, Lys, His, Arg, and Pro [42]. However, we found Arg, His, Met, Leu, and Thr in the serum were significantly decreased as the dietary valine increased in this study. In the post-peak period of laying hens, Azzam et al. found that serum free Ile, Phe, Tyr, and Val were significantly decreased as supplemental L-Thr increased [43]. Similarly, Eder and Peganova’s report indicated that the concentration of Lys in plasma decreased with increasing dietary isoleucine concentration [14]. These differences may be explained by decreasing serum levels of these AAs in response to increasing dietary Val levels, which can indicate a greater utilization of these AAs.

In the small intestines, dietary protein can be absorbed in the form of amino acids or, more marginally, dipeptides or tripeptides by peptide and amino acid transporters. PepT1, a peptide transporter, can transport di- and tripeptides from the intestinal lumen into enterocytes [18]. In the current study, the mRNA expression levels of PepT1 in the duodenum, jejunum, and ileum showed quadratic decreases as the dietary valine levels increased. Duodenum and jejunum are the main absorption sites of valine, and PepT1 has the highest expression levels in the duodenum, followed by the jejunum and ileum [44,45,46]. In Caco-2 cells, it has been confirmed that oxidative injury can inhibit PepT1 transport velocity [27]. However, Jiang et al. reported that dietary threonine supplementation did not affect the expression levels of the PepT1 in the duodenum or ileum of Chinese yellow-feathered embryonic chicks [47]. Moreira Filho et al. found that in ovo feeding with threonine increased the mRNA expression level of PepT1 in the ileum of broiler chicks on the day of hatching, but threonine had no effect on the expression of PepT1 in chicks aged 21 days [48]. Together with increased serum MDA levels and decreased egg quality, it may be indicated that oxidative stress inhibits the absorption of protein, resulting in the reduction of egg quality.

B^0^AT1 is responsible for the uptake of neutral amino acids such as Met, Leu, Ile, and Val [19]. We found the mRNA expression level of B^0^AT1 was quadratically decreased in the jejunum and ileum as the dietary valine levels increased, whereas it had no effect on the duodenal expression levels. It has been demonstrated that B^0^AT1 is mainly expressed in the intestinal villi, and the expression level was gradually increased from duodenum to ileum [49,50,51]. Dietary tryptophan supplementation upregulated the gene expression level of B^0^AT1 in Chinese broiler breeders, whereas dietary threonine supplementation did not affect the B^0^AT1 expression levels in the duodenum or ileum of Chinese yellow-feathered embryonic chicks [47,51]. The B^0^AT transporters can transport neutral and cationic amino acids including Gly, Ser, Thr, Cys, Tyr, Asn, Gln, His, Lys, and Arg [19]. We found the serum free Arg, His, Thr, Gly, and Ser showed quadratically decreases with the increase of dietary valine concentration, which agrees with previous reports [42].

SNAT2, a Na+-coupled neutral amino acid transporter, mainly expressed in the small intestine, and up-regulation of SNAT2 expression level represented an increase in the capacity of amino acid transportation [20]. In the current study, the mRNA expression of SNAT2 in the jejunum showed a quadratic decrease with the increase of dietary valine concentration, but did not affect the expression levels of duodenal and ileal. The previous studies indicated that a higher expression of SNAT2 contributed to the absorbance of BCAAs, which resulted in the activation of the protein synthesis signaling pathway [25]. SNAT2 is ubiquitously expressed and transported by L-glutamine, and this transport process is highly energized, so that Glu, Gly, Pro, and Ala reach high transmembrane gradients and constitute major components of the intracellular amino acid pool [52]. Consistently, our results revealed that the expression levels of SNAT2 in the jejunum and the serum free Glu, Gly, and Ala levels all showed quadratic decreases as the dietary valine concentration increased, which may result in poor egg quality.

ATB^0,+^ is an Na+-dependent neutral and cationic amino acid transporter and was first found in mouse blastocysts [21]. Our results suggest that with the increase of dietary valine concentration, the mRNA expression level of ATB^0,+^ in the jejunum displays a quadratic increase but did not affect the expression levels in the duodenum and ileum. ATB^0,+^ can transport L-enantiomers of neutral and cationic amino acids as well as D-enantiomers such as D-Ser, D-Ala, D-Met, D-Leu, and D-Trp [53]. Our results indicated that dietary valine treatment significantly increased the concentrations of serum free Ile, Lys, Phe, Val, and Tyr. However, serum free Ser, Ala, Met, and Leu in the current study showed significantly decreases with the increase of dietary valine concentration, which is inconsistent with Hatanta et al.’s study [53]. Our results in the current study may indicate that higher expression levels of ATB^0,+^ in the jejunum may help the epithelial cells to absorb and transport amino acids from the intestinal lumen.

LAT1 and LAT4, L-type amino acid transporters, can deliver a narrow range of neutral amino acids into cells, including Leu, Isoleu, Val, Phe, and Met [24,54]. In the present study, the mRNA expression levels of LAT1 showed linear or quadratic decreases in the jejunum and ileum with the increase of dietary valine concentration. In the chickens, levels of LAT1 were higher in the ileum 14 days post-hatching [23], and the Eimeria praecox challenge can cause down-regulation in the duodenum and ileum [55]. Supplemented melatonin increased the LAT1 expression level in the jejunum of laying hens during the laying peak period compared to the pre-laying or the post-laying period [43]. With the increase of dietary valine levels, we found the mRNA level of LAT4 in the jejunum was quadratically increased, whereas it was significantly decreased in the duodenum, but it. did not affect the expression level of the ileum. In the intestine, LAT4 is mainly present in the cells of the crypt and expressed in the basolateral membrane [24,56]. At the basolateral membrane of enterocytes, LAT4 has been shown to mediate the transport of Phe, Leu, Isoleu, and Met, contributing to the efflux of amino acids after their luminal uptake from the intestinal lumen [23,57]. Consistently, our serum free amino acids revealed that Leu, Phe, and Met were significantly decreased with the increase of dietary valine supplementation, whereas the serum Ile was significantly increased. It has been demonstrated that LAT4 was localized in the basolateral epithelia and was necessary to mediate a balance of its amino acid substrates between the extracellular space and the cytosol [57]. In the hypothalamus, LAT1 and LAT4 could putatively act as amino acid exchangers, were involved in the transport of L-3,4-dihydroxyphenylalanine across the blood–brain barrier, and also play a role in neuronal cell proliferation in the brain [58].

## 5. Conclusions

This study revealed that valine feeding in laying hens could act locally on the intestine, enhance the intestinal digestive enzymes secretion, and promote the expression of jejunal ATB^0,+^, LAT4, and corresponding AA uptake and utilization, and result in improved egg-laying performance. The optimal dietary valine concentration that maximized the laying rate for Fengda No.1 laying hens aged from 33 to 41 weeks was 0.79% according to quadratic regression analysis. It is worth noting that dietary supplementation with high levels of valine may have detrimental effects on egg quality, immune function, and the absorption of nutrients during the peak production in layers.

## Figures and Tables

**Table 1:** Composition and nutrient levels of the basal diet (air-dry basis).

Ingredients	Dietary Valine Levels (%) ^a^				
	0.59	0.64	0.69	0.74	0.79
Corn	66.6	66.6	66.6	66.6	66.6
Soybean meal	10.5	10.65	11.2	11.55	11.8
Wheat bran	2.9	2.9	2.91	2.92	2.92
Peanut meal	8.7	8.5	7.9	7.5	7.2
Limestone	9.3	9.3	9.3	9.3	9.3
Soybean oil	0.3	0.3	0.3	0.3	0.3
DL-Methionine (98%)	0.16	0.16	0.15	0.15	0.15
Lysine (78%)	0.11	0.11	0.11	0.1	0.1
Valine (98%)	0	0.0508	0.1016	0.1523	0.2031
CaHPO4	0.6	0.6	0.6	0.6	0.6
Salt	0.36	0.36	0.36	0.36	0.36
Choline chloride, 60%	0.2	0.2	0.2	0.2	0.2
Mineral and vitamin premix ^b^	0.27	0.27	0.27	0.27	0.27
Calculated nutritional level, %					
Crude protein (CP)	14.7	14.7	14.7	14.7	14.7
ME, MJ/Kg	2.68	2.68	2.68	2.68	2.68
Analyzed nutrients					
Crude protein (CP)	14.65	14.72	14.75	14.74	14.78
Calcium	3.58	3.58	3.59	3.59	3.59
Total phosphorus	0.46	0.46	0.46	0.46	0.46
Methionine	0.36	0.36	0.36	0.37	0.37
Lysine	0.66	0.66	0.66	0.67	0.67
Threonine	0.48	0.48	0.48	0.49	0.49
Tryptophan	0.14	0.14	0.14	0.14	0.14
Arginine	1.05	1.04	1.03	1.03	1.02
Valine	0.59	0.64	0.69	0.74	0.79

^a^ Analyzed value of valine in pooled experimental diets were 0.59, 0.64, 0.69, 0.74, and 0.79%. ^b^ The premix provided the following per kilogram of diet: vitamin A, 7500 IU; vitamin D3, 2500 IU; vitamin E, 49.5 mg; vitamin K3, 2.5 mg; vitamin B1, 1.5 mg; vitamin B2, 4 mg; vitamin B6, 2 mg; vitamin B12, 0.02 mg; niacin, 30 mg; folic acid, 1.1 mg; pantothenic acid, 10 mg; biotin, 0.16 mg; chloride choline, 400 mg; Sodium chloride, 2500 mg; Fe, 80 mg; Cu, 20 mg; Mn, 60 mg; Zn, 80 mg; I, 0.8 mg.

**Table 2:** Primers used for quantitative real-time PCR.

Gene Name	Primer	Primer Sequence (5′-3′)	Accession No.
β-Actin	Forward	TCCCTGGAGAAGAGCTATGAA	NM_205518.1
	Reverse	CAGGACTCCATACCCAAGAAAG	
PepT1	Forward	CTTGGCAGATCCCTCAGTATTT	XM_034074354.1
	Reverse	GTTGGGCTTCAACCTCATTTG	
B^0^AT1	Forward	CATGATCGGACACAAGCCCA	XM_419056.6
	Reverse	AGCATAGACCCAGCCAGGATA	
ATB^0,+^	Forward	TAAACCAGTGCAATTTCCCA	XM_001199603.1
	Reverse	CGATGTTGCCAGTCTCATC	
SNAT2	Forward	GAAGGAGTTCAGTTGGTGGCG	NM_001305439.1
	Reverse	CGGATAGTAGGGACAAAGATAACGAG	
LAT1	Forward	TGGCCTTGTAC AGTGGTCTT	NM_001030579.2
	Reverse	GCTTCGGACTTC AGC ATCTG	
LAT4	Forward	ACAACTGTGGGACGCCGACTGA	XM_415803.6
	Reverse	GGCATTGGTGGCATTGGTGATTT	

β-Actin, beta-actin; PepT1, solute carrier family 15 members 1; B^0^AT1, solute carrier family 6 members 19; ATB^0,+^, solute carrier family 6 members 14; SNAT2, solute carrier family 38 members 2; LAT1, solute carrier family 7 members 5; LAT4, solute carrier family 43 members 2.

**Table 3:** Effects of dietary valine levels on productive performance of laying hens.

Item	Dietary Valine Levels (%)					SEM	*p*-Value	
	0.59	0.64	0.69	0.74	0.79		L	Q
Egg mass (a/repeat/d)	25.99 ^ab^	25.87 ^b^	26.23 ^ab^	26.31 ^ab^	26.43 ^a^	0.14	0.003	0.012
Laying rate (%) ^1^	81.63	81.28	82.46	82.52	82.86	0.43	0.007	0.026
Egg weight (g)	48.50 ^a^	48.50 ^a^	48.28 ^b^	48.31 ^ab^	48.27 ^b^	0.08	0.011	0.034
Broken egg rate (%)	0.39 ^a^	0.24 ^ab^	0.24 ^ab^	0.24 ^ab^	0.13 ^b^	0.05	0.003	0.011
Feed intake (g/hen/d)	100.90	101.20	100.45	100.83	100.34	2.30	0.835	0.977
FCR (kg feed/kg egg)	2.55 ^a^	2.56 ^a^	2.52 ^bc^	2.53 ^b^	2.51 ^c^	0.02	0.001	0.003

Values are represented as the mean and SEM (n = 32). Means with different superscript letters (a–c) within a column differ significantly (*p* < 0.05). FCR, feed conversion ratio. ^1^ Y = 81.451 + 0.293X + 0.019X^2^; R^2^ = 0.004, *p* = 0.026. This equation yielded an optimized total dietary valine concentration of 0.79%.

**Table 4:** Effects of dietary valine levels on egg quality of laying hens.

Item		Dietary Valine Levels (%)					SEM	*p*-Value	
		0.59	0.64	0.69	0.74	0.79		L	Q
Egg weight, g		50.32	51.03	50.34	49.55	49.28	0.67	0.095	0.180
Eggshell strength (N)		4.88	4.58	4.65	4.75	4.80	0.15	0.944	0.417
Eggshell thickness (mm^−2^)	Sharp end	0.42 ^ab^	0.42 ^ab^	0.41 ^ab^	0.43 ^a^	0.40 ^b^	0.005	0.024	0.048
	Equator	0.40	0.40	0.39	0.41	0.38	0.005	0.370	0.143
	Blunt end	0.39	0.38	0.38	0.39	0.38	0.006	0.473	0.774
Albumen height (mm)		5.19 ^ab^	5.78 ^a^	5.18 ^ab^	5.09 ^ab^	4.67 ^b^	0.20	0.010	0.006
Haugh unit		74.45 ^ab^	77.23 ^a^	74.73 ^ab^	71.45 ^ab^	69.40 ^b^	1.85	0.011	0.014
Egg yolk color (points)		10.34 ^a^	10.43 ^a^	10.53 ^a^	10.43 ^a^	9.07 ^b^	0.13	<0.001	<0.001

Values are represented as the mean and SEM (pooled) (n = 30). Means with different superscript letters (a, b) within a column differ significantly (*p* < 0.05).

**Table 5:** Effects of dietary valine levels on serum antioxidant capacity of laying hens.

Serum	Dietary Valine Levels (%)					SEM	*p*-Value	
	0.59	0.64	0.69	0.74	0.79		L	Q
CAT, U/mL	8.19 ^ab^	6.59 ^ab^	11.55 ^a^	7.24 ^ab^	5.74 ^b^	1.71	0.229	0.041
T-SOD, U/mL	175.79	175.33	187.55	175.85	166.15	7.46	0.544	0.255
T-AOC, U/mL	0.90 ^ab^	0.92 ^ab^	0.76 ^b^	0.85 ^ab^	1.02 ^a^	0.05	0.312	0.025
GSH-Px, U/mL	274.29	180.57	278.79	278.06	259.71	15.19	0.229	0.488
MDA, nmol/mL	2.97 ^ab^	2.90 ^ab^	2.49 ^b^	3.14 ^ab^	4.80 ^a^	0.40	0.029	0.002

Values are represented as the mean and SEM (pooled) (n = 8). Means with different superscript letters (a, b) within a column differ significantly (*p* < 0.05). GSH-Px, glutathione peroxide; SOD, superoxide dismutase; T-AOC, total antioxidant capacity; CAT, catalase; MDA, malondialdehyde.

**Table 6:** Effects of dietary valine levels on serum free amino acids of laying hens.

Amino Acids, mg/L	Dietary Valine Levels (%)					SEM	*p*-Value	
	0.59	0.64	0.69	0.74	0.79		L	Q
Essential AA								
Arginine	102.01 ^b^	99.83 ^c^	107.65 ^a^	103.65 ^b^	96.40 ^d^	0.41	0.244	0.001
Histidine	2.09	2.09	1.91	1.85	1.87	0.05	0.002	0.008
Isoleucine	10.90 ^d^	11.73 ^c^	12.67 ^b^	13.33 ^ab^	13.86 ^a^	0.18	<0.001	<0.001
Leucine	19.29 ^ab^	18.90 ^b^	19.75 ^a^	17.76 ^c^	18.71 ^b^	0.14	0.042	0.134
Lysine	32.81 ^b^	32.98 ^b^	33.69 ^b^	35.15 ^a^	35.75 ^a^	0.28	<0.001	<0.001
Methionine	8.89 ^a^	8.30 ^b^	7.98 ^c^	7.45 ^d^	7.17 ^e^	0.05	<0.001	<0.001
Phenylalanine	17.53 ^b^	17.17 ^b^	18.52 ^a^	17.46 ^b^	18.77 ^a^	0.18	0.011	0.032
Threonine	50.65 ^a^	48.80 ^c^	49.93 ^a^	46.96 ^b^	50.06 ^a^	0.15	0.169	0.019
Valine	13.02 ^e^	13.90 ^d^	15.06 ^c^	16.68 ^b^	18.14 ^a^	0.17	<0.001	<0.001
Nonessential AA								
Alanine	81.67 ^a^	78.50 ^b^	76.12 ^c^	75.08 ^c^	78.40 ^b^	0.32	0.005	<0.001
Aspartic acid	2.58 ^a^	2.41 ^d^	2.45 ^cd^	2.49 ^bc^	2.52 ^ab^	0.01	0.169	0.019
Cystine	24.56 ^b^	22.31 ^c^	26.36 ^a^	21.03 ^d^	23.60 ^b^	0.22	0.304	0.598
Glutamic acid	31.00 ^b^	33.59 ^a^	32.41 ^ab^	31.47 ^b^	30.57 ^b^	0.29	0.165	0.004
Glycine	56.67 ^a^	51.47 ^c^	53.74 ^b^	49.39 ^d^	52.04 ^c^	0.26	0.002	<0.001
Proline	16.61	16.30	17.01	16.40	16.58	0.30	0.966	0.962
Serine	54.02 ^a^	52.98 ^b^	51.31 ^c^	50.70 ^c^	50.32 ^c^	0.26	0.165	0.004
Tyrosine	34.17 ^a^	30.20 ^c^	32.30 ^b^	33.26 ^ab^	33.49 ^a^	0.25	0.480	0.015

Values are represented as the mean and SEM (n = 4). Means with different superscript letters (a–e) within a column differ significantly (*p* < 0.05).

## Data Availability

The data presented in this study are available on request from the corresponding author.
